# After microvascular decompression to treat trigeminal neuralgia, both immediate pain relief and recurrence rates are higher in patients with arterial compression than with venous compression

**DOI:** 10.18632/oncotarget.14765

**Published:** 2017-01-20

**Authors:** Lei Shi, Xiaoyan Gu, Guan Sun, Jun Guo, Xin Lin, Shuguang Zhang, Chunfa Qian

**Affiliations:** ^1^ Department of Neurosurgery and Anesthesiology, The First People’s Hospital of Kunshan affiliated with Jiangsu University, Suzhou, P. R. China; ^2^ Department of Rehabilitation, The 454th Hospital of Chinese PLA, Nanjing, Jiangsu, China; ^3^ Department of Neurosurgery, Fourth Affiliated Yancheng Hospital of Nantong University, Yancheng, P. R. China; ^4^ Department of Neurosurgery, Affiliated Nanjing Brain Hospital, Nanjing Medical University, Nanjing, P. R. China

**Keywords:** trigeminal neuralgia, microvascular decompression, arterial compression, venous compression, recurrence

## Abstract

We explored differences in postoperative pain relief achieved through decompression of the trigeminal nerve compressed by arteries and veins. Clinical characteristics, intraoperative findings, and postoperative curative effects were analyzed in 72 patients with trigeminal neuralgia who were treated by microvascular decompression. The patients were divided into arterial and venous compression groups based on intraoperative findings. Surgical curative effects included immediate relief, delayed relief, obvious reduction, and invalid result. Among the 40 patients in the arterial compression group, 32 had immediate pain relief of pain (80.0%), 5 cases had delayed relief (12.5%), and 3 cases had an obvious reduction (7.5%). In the venous compression group, 12 patients had immediate relief of pain (37.5%), 13 cases had delayed relief (40.6%), and 7 cases had an obvious reduction (21.9%). During 2-year follow-up period, 6 patients in the arterial compression group experienced recurrence of trigeminal neuralgia, but there were no recurrences in the venous compression group. Simple artery compression was followed by early relief of trigeminal neuralgia more often than simple venous compression. However, the trigeminal neuralgia recurrence rate was higher in the artery compression group than in the venous compression group.

## INTRODUCTION

Trigeminal neuralgia is characterized by pain along the anatomical areas innervated by the trigeminal nerve [1]. The cause of trigeminal neuralgia is believed to be due to vascular compression of the trigeminal nerve [2]. Cranial magnetic resonance tomographic angiography (MRTA) is commonly used to diagnose trigeminal neuralgia, but the symptoms of the disease and the experience of doctors are considered to be more important [3]. With the development of microsurgical techniques, microvascular decompression (MVD) has obtained good curative effects in the treatment of trigeminal neuralgia. Microsurgical decompressions confirmed that vascular compression on the trigeminal nerve is correlated with trigeminal neuralgia [4]. Artery compression has been more researched, but trigeminal neuralgia can also be caused by venous pressure [5]. This study explored the influences of both arterial and venous compression on prognosis of trigeminal neuralgia after microvascular decompression.

## RESULTS

### The source of vascular compression varied in both groups

Of the 40 patients with arterial compression, the trigeminal nerve was compressed by the superior cerebellar artery in 21 cases, the anterior inferior cerebellar artery in 13 cases, the posterior inferior cerebellar artery in 3 cases, the vertebral artery in 1 case, and by unknown-derived vessels in 2 cases. Venous compression of the trigeminal nerve was relatively complex. Of the 32 patients with venous compression, the trigeminal nerve was compressed by the pons-transverse vein in 13 cases, the pons-trigeminal vein in 7 cases, the cerebellopontine fissure in 5 cases, the middle cerebellar peduncle vein in 3 cases, the petrosal vein in 3 cases, and by an unnamed vein in 1 case.

### Arterial compression group had a higher rate of immediate pain relief

In the arterial compression group, 32 cases had immediate relief of postoperative pain (80.0%), 5 cases had delayed relief (12.5%), and 3 cases had an obvious reduction (7.5%). In the venous compression group, 12 cases had immediate relief of postoperative pain (37.5%), 13 cases had delayed relief (40.6%), and 7 cases had an obvious reduction (21.9%). Immediate pain relief was greater in the arterial compression group than the venous compression group (P=0.00, χ2 analysis). After operation in the arterial compression group, 6 cases (15.0%) were accompanied with facial numbness and superficial hypoesthesia, 1 case (2.5%) had hearing loss, and 1 case (2.5%) had temporary facial paralysis. After operation in the venous compression group, 5 cases (15.6%) were accompanied with facial numbness and superficial hypoesthesia, 3 cases (9.38%) had hearing loss, and 3 cases (9.38%) had temporary facial paralysis. Statistical analysis showed no difference in facial numbness (P=0.942 by χ2 analysis), hearing loss (P=0.317 by Fisher analysis), or temporary facial paralysis (P=0.317 by Fisher analysis) between the two groups.

### Trigeminal neuralgia recurrence rate is lower in venous compression group than arterial compression group

In the arterial compression group of 40 patients, 37 cases agreed to follow up for 2 years, and 3 cases were missed during the first year of follow up. Trigeminal neuralgia recurred in 6 cases, which is a recurrence rate of 17.6%. Among the 6 cases, 2 cases in this study had severe pain and underwent the operation again, and the other 4 cases had mild pain so they chose an oral carbamazepine treatment. In the venous compression group of 32 patients, 29 cases agreed to follow up, 3 cases were missed during the first year of follow up, and there were no cases of trigeminal neuralgia recurrence. Fisher analysis showed a difference between the two groups (P=0.036), which implied that the long-term recurrence rate in the venous compression group is lower than that in the arterial vascular compression group.

## DISCUSSION

Vascular compression is the main pathophysiology mechanism of trigeminal neuralgia. When the compression of blood vessels on the nerve root accumulates, demyelinating change of the nerve root directly affects the afferent impulse of trigeminal nerve fibers [6, 7]. This change activates the negative feedback in vivo to reduce the threshold of pain-sensitive nerve endings (receptors) to increase the afferent impulse, and make up for the interference brought about by the demyelinating changes [8]. But with the decreased threshold of nerve endings, the nerve endings are in a hypersensitive state, and even a slight stimulus can also cause the rapid increase of afferent impulse, thus eventually forming the feeling of pain [9].

Microvascular decompression is the first choice of treatment for trigeminal neuralgia [10, 11]. The responsible blood vessels can include the superior cerebellar artery, anterior inferior cerebellar artery, posterior inferior cerebellar artery, to vertebral basilar artery, where superior cerebellar artery and its branches are the most common [12]. Venous compression is not as common as arterial compression in the trigeminal nerve, but it is not rare either [13]. Matsushima et al. reported that among 121 cases of trigeminal neuralgia, veins accounted for 20%, including simple venous compression for 5.7% [14].

Among the 40 cases in this study with simple artery compression, pain was immediately relieved in 32 cases, which is an early remission rate of 80.0%. Of the 32 cases with simple venous compression, pain was immediately relieved in only 12 cases, an early remission rate of 37.5%. It can be concluded that patients with trigeminal neuralgia caused by simple artery compression saw more immediate pain relief after microvascular decompression than those patients whose neuralgia was caused by simple venous compression.

The most common postoperative complications included facial hypoesthesia and hearing loss, while temporary facial paralysis, cerebrospinal fluid leakage, brain stem or cerebellum infarction or hemorrhage and ataxia were also reported. Most cranial nerve injuries are mild, and can gradually resolve. This study found there are more patients with facial numbness, superficial hypoesthesia, hearing loss, and temporary facial paralysis in the artery compression group than in the venous compression group. The cause may be that venous compression is often closely related with the trigeminal nerve, so its separation process has more operations than simple artery compression, thus causing wider range of surgical imaging of trigeminal nerve. Literature reported postoperative recurrence of MVD was 3% ~ 20%, and the recurrence often occurred 2 years after the operation [15]. This study found that the recurrence rate of trigeminal neuralgia was 17.6% in the arterial compression group, while there was no recurrence in the venous compression group.

## CONCLUSIONS

While patients with trigeminal neuralgia caused by simple artery compression reported more immediate pain relief after microvascular decompression compared to patients whose neuralgia was caused by simple venous compression, the long-term recurrence rate of the latter was lower than that of the former. There were no differences in postoperative complications between the two groups.

## MATERIALS AND METHODS

### Patients and informed consent

This study included 40 male and 32 female cases, and the average age was 63.7 ± 6.60 years old. There were 56 cases above 60 y, and the disease course was 3 - 16 y. Patients of the arterial compression group all showed lateral facial paroxysmal pain, including first branch pain in 6 cases, second branch pain in 9 cases, third branch pain in 7 cases, 1+2 branches pain in 5 cases, 2+3 branches pain in 10 cases, and all three branches pain in 3 cases. Patients of venous compression group all showed lateral facial paroxysmal pain, including first branch pain in 3 cases, second branch pain in 10 cases, third branch pain in 6 cases, 1+2 branches pain in 3 cases, 2+3 branches pain in 8 cases, and all three branches pain in 2 cases.

All patients were given surgical treatment after failed preoperative treatments including oral administration of carbamazepine or acupuncture, etc. All compressions of the trigeminal nerve were due to simple arterial or venous compression. The study was approved by the Ethics Committee of each hospital. Written informed consent was obtained from all the patients prior to surgery, and all the patients consented to the submission of this report for publication.

### Operation method

Microvascular decompressions were all carried out via a routine suboccipital retrosigmoid approach. Cerebrospinal fluid was drained slowly under a microscope, and when the cerebellum collapsed, the area was explored. Adhesion between arachnoid and cranial nerves was sharply separated and fully released. The arteries responsible for the compression were found, and Teflon cotton was placed to block the blood vessel in the brain cistern of the trigeminal nerve.

**Figure 1:**
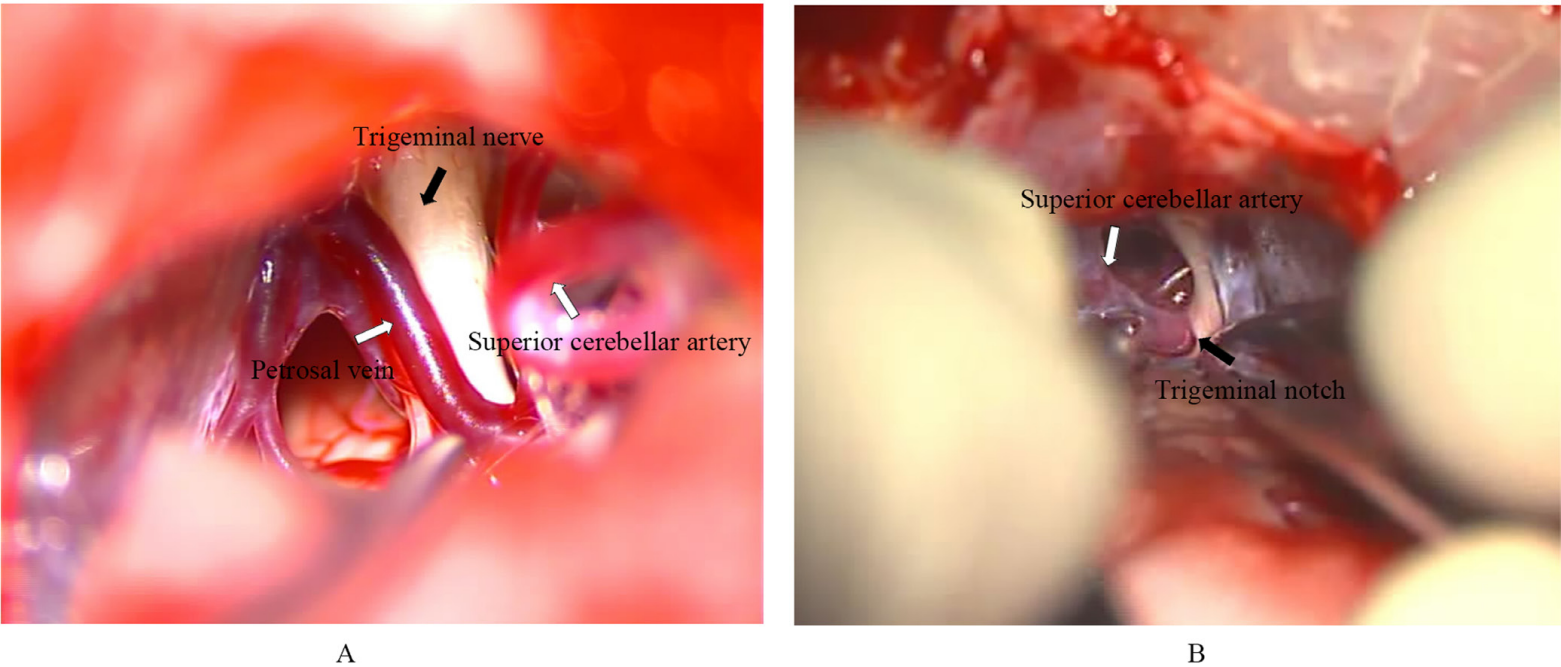
Intraoperative photographs of trigeminal nerve lysis **A**. Trigeminal nerve compressed by petrosal vein. **B**. Trigeminal nerve compressed by superior cerebellar artery.

For the larger responsible veins, especially the trunk of super petrosal vein complex, they were retained. Small veins and branches of super petrosal veins were blocked with electric coagulation, adhesion, and compression to withdraw venous compression, and then the same operation was carried out between the responsible veins and the trigeminal nerve. None of the patients were given drainage tubes, and the meninges were sutured tightly. Dura mater defect was repaired with autologous fascia, and the scalp was sutured. Intraoperative photographs of trigeminal nerve lysis is shown in Figure 1.

### Postoperative observation and follow-up

Recovery from trigeminal neuralgia was regularly inspected and recorded after operation, and the improvement was divided into four types: (a) Postoperative pain was relieved immediately, and entirely disappeared within 1 day after the operation; (b) pain relief was delayed, and postoperative pain gradually decreased to zero within 1 month; (c) the pain decreased, but still a small dose of carbamazepine was needed to control pain after operation; (d) the pain was not obviously improved. Recurrence referred to the repetition of pain after a period of complete relief.

### Statistical methods

SPSS 16.0 software was used for the statistical analysis. For the data analysis, the mean ± standard deviation was employed. We used the χ2 or Fisher analysis to test associations between categorical variables, and the t-test for continuous variables. *P* < 0.05 was considered statistically significant.
